# Female Genital Mutilation in Ghana: Prevalence and Socioeconomic Predictors

**DOI:** 10.1155/2021/6675579

**Published:** 2021-05-11

**Authors:** Abdul Rauf Alhassan, John Nyaaba Anyinzaam-Adolipore

**Affiliations:** ^1^Department of Surgery, Tamale Teaching Hospital, P.O. Box TL 16 Tamale, Ghana; ^2^Department of supervision/inspectorate unit, Ghana education service, P.O. Box KA 20 Karaga, Ghana

## Abstract

**Background:**

Each year, not less than three million women are circumcised, and more hundred million females have already been circumcised. In many African societies, the practice of female genital mutilation (FGM) is a serious cultural practice. Aim: This current study is aimed at identifying the socioeconomic predictors of female genital mutilation in Ghana.

**Methods:**

The design adopted for this study was a descriptive cross-sectional survey relying on data from the Ghana Multiple Indicator Cluster Survey (MICS) 2017/18. SPSS software was used for data analysis. Chi-square and binary logistic regression were used for associations.

**Results:**

Overall FGM prevalence, this study recorded was 11.7%. The region with the highest (50.5%) prevalence was the Upper West Region. Area of residence predicted rural (AOR = 2.30, 95%C.I. = 1.75–3.00) Upper West/western Region (AOR = 1.84, 95%C.I. = 1.23–2.75). In terms of ethnicity, the tribes that predicted FGM when compared with the Akan tribe were Guan (AOR = 8.91, 95%C.I. = 3.53–22.51), Gruma (AOR = 6.45, 95%C.I. = 2.91–14.31), Mole-Dagbani (AOR = 38.10, 95%C.I. = 21.20–68.49), Grusi (AOR = 45.30, 95%C.I. = 24.47 − 83.49), Mande (AOR = 68.58, 95%C.I. = 30.85 − 152.42), and other tribes (AOR = 29.33, 95%C.I. = 16.11–53.39). Women in the richest/poorest wealth index quintile (AOR = 1.80, 95%C.I. = 1.19–2.72).

**Conclusion:**

The study prevalence of FGM is still high in the northern part of Ghana, and the predicted factors were residence region, ethnicity, educational level, and economic status.

## 1. Introduction

Female genital mutilation (FGM) is defined by the World Health Organization (WHO) as the practice of cutting parts or complete removal of female external genitalia for culture medicine. It was important to classify FGM as it is significant for clinical practice, management, recording, and reporting of prevalence. FMG is categorized into four and further subdivided into subtypes [1].

In many African societies, the practice of female genital mutilation (FGM) is a serious cultural practice. FGM practice can be traced in 28 African nations, even though the use of nationality as a predictor is less significant as compared to the traditional culture. Statistics have it that each year, not less than three million women are circumcised, and more hundred million females have already been circumcised [2].

In Ghana, the magnitude of FGM practice is variable among regions and districts, but this practice is very dominant among the northern tribes [3, 4]. Even though the overall prevalence was 4%, that of the Upper East Region was 38%, and that of a district (Bawku municipality) was 82% [4]. It is believed that the higher prevalence in northern Ghana can be attributed to the variable nature of people and culture and the proximity of neighboring nations such as Mali, Togo, and Burkina Faso where the practice is widespread [5].

There are a lot of short- and long-term complications associated with the practice of FGM. Some of these short-period complications include psychological trauma, hemorrhage, shock, very severe pain, genital tissue swelling, impaired wound healing, increased exposure to blood, and transmittable infections such as human immunodeficiency virus (HIV) and hepatitis B sharing of the same knife or bladder during the procedure [6]. Moreover, among the long-term complications are chronic infections of the genital tract, reproductive tract and urinary tract, menstrual problems, and sexual performance disturbances such as pain during sexual intercourse [6–9].

Many factors have shown a significant relationship with FGM practice, and among these include demographic factors such as age, educational background, religion, culture, and economic status [10–12]. In sub-Saharan African nations, studies on economic status had a significant relation with FGM [10, 11]. Women from better economic homes were less likely to involve in FGM. And younger and well-educated women are negatively associated with FGM practice [10–13].

Additionally, a study in northern Ghana found a significant relationship between a woman's demographic characteristics and FGM. Predictors' factors were aged 35–49 years, no formal education or primary education, and married women [5]. This current study is aimed at identifying the socioeconomic predictors of female genital mutilation in Ghana.

## 2. Materials and Methods

The design adopted for this study was a descriptive cross-sectional survey relying on data from the Ghana Multiple Indicator Cluster Survey (MICS) 2017/18. The survey was conducted from October 2017 to January 2018 by the Ghana Statistical Service with collaborator partners such as the Ministry of Health, Ministry of Education, Ministry of Sanitation and Water Resources, Ministry of Gender, Children, and Social Protection, Ghana Health Service, and the Ghana Education Service as part of the Global MICS Programme and technical support from the United Nations Children's Fund (UNICEF), with government funding and financial support from UNICEF, KOICA, UNDP, USAID, and the World Bank through the Statistics for Results Facility–Catalytic Fund (SRF-CF).

The sampling frame assumed was from the Ghana 2010 Population and Housing Census (PHC). This encompassed all women (10,840) aged 15-49 years who were permanent occupants of selected households or visitors who stayed in selected households the night before the survey.

### 2.1. Statistical Analysis

SPSS version 20 (IBM Corp., 2011, and NY) was used for the analysis. Categorical variables results were presented using frequencies and percentages in tables. The association between dependent and independent variables was done using chi-square. The binary logistic regression model was used to identify the predictor variables of FGM. Statistical significance was set at a *P* value of <0.05.

## 3. Results

### 3.1. Socioeconomic Characteristics of Respondents

The majority (56.6%) of the women were from rural, and dominant (15.6%) of them were from the Ashanti region and in terms of ethnicity, about 37.2% of them were from the Akan tribe. Most (73.1%) of them first got married when they were between 15 and 24 years. About 37.8% of them had a preprimary educational level or no education. Health insurance coverage was 55.6% among the respondents, and 29.7% of them were in the poorest wealth index quintile. The majority (88.8%) of them had no functional difficulty (Table 1).

### 3.2. Female Genital Mutilation Prevalence in Ghana and Associated Factors

Overall FGM prevalence, this study recorded was 11.7%. The region with the highest (50.5%) prevalence was the Upper West Region, and the lowest (0.9%) prevalence was recorded in the central and eastern regions (Table 2).

Chi-square analysis revealed a significant relationship between FGM and socioeconomic characteristics such as area of residence, the region from, age at first marriage, ethnicity, educational level, health insurance coverage, functional difficulty, and wealth index quintile (*P* ≤ 0.001) (Table 3).

### 3.3. Predictors of FGM in Ghana

Area of residence predicted FGM, and those from rural were more likely 2.3 times to engage in FGM compared to those from urban (AOR = 2.30, 95%C.I. = 1.75–3.00). Women from all regions except the Upper West Region when compared to those from the western region were protected from FGM (*P* < 0.05). Women from the Upper West Region were more likely 1.8 times to engage in FGM compared to those from the western region (AOR = 1.84, 95%C.I. = 1.23–2.75). In terms of ethnicity, the tribes that predicted FGM when compared with the Akan tribe were Guan (AOR = 8.91, 95%C.I. = 3.53–22.51), Gruma (AOR = 6.45, 95%C.I. = 2.91–14.31), Mole-Dagbani (AOR = 38.10, 95%C.I. = 21.20–68.49), Grusi (AOR = 45.30, 95%C.I. = 24.47 − 83.49), Mande (AOR = 68.58, 95%C.I. = 30.85 − 152.42), and other tribes (AOR = 29.33, 95%C.I. = 16.11–53.39).

The history of FGM was less likely 0.8 times among women with a marriage age of 15-24 years when compared to those with marriage age before 15 years (AOR = 0.75, 95%C.I. = 0.62–0.91). Women with educational level primary and above were less likely to engage in FGM compared to those with preprimary or none (*P* < 0.05). Women from the richest wealth index quintile were more likely 1.8 times to engage in FGM compared to those from the poorest wealth index quintile (AOR = 1.80, 95%C.I. = 1.19–2.72) (Table 4).

## 4. Discussion

Overall FGM prevalence, this study recorded was 11.7%. The region with the highest (50.5%) prevalence was the Upper West Region, and the lowest (0.9%) prevalence was recorded in the central and eastern regions. There is a decline in national prevalence when compared to an earlier study using MICS data, which recorded a prevalence of 15.9%. In this same study, the prevalence was high among women of the Upper West Region [14]. This confirms other results that in Ghana, the magnitude of the practice of FGM is variable among regions and districts, and that this practice is very dominant among the northern tribes [3, 4]. Even within the regions, it is variable among districts in a study through the overall prevalence was 4%, and that of the Upper East region was 38%, and that of a district (Bawku municipality) was 82% [4]. The variation of prevalence across the regions of Ghana can be attributed to the variable cultural practices, and this confirms the fact that using nationality as a predictor is less significant when compared with using traditional culture [2]. It is believed that the higher prevalence in northern Ghana can be attributed to the variable nature of people and culture and the proximity of neighboring nations such as Mali, Togo, and Burkina Faso where the practice is widespread [5].

This is the first of a few epidemiological studies on FGM with an effort of shedding light on etiology and predictors. This study was not without limitations as not all variables significantly related to the study topic were studied, for example, religious affiliation. Two variable analyses revealed a significant relationship between FGM and socioeconomic characteristics such as area of residence, the region from, age at first marriage, ethnicity, educational level, health insurance coverage, functional difficulty, and wealth index quintile (*P* ≤ 0.001). These factors were significantly related to FGM in other studies [10–12].

Area of residence predicted FGM, and those from rural were more likely 2.3 times to engage in FGM compared to those from urban. Women from the regions except the Upper West Region were protected from FGM when compared to those from the western region (*P* < 0.05). Women from the Upper West Region were more likely 1.8 times to engage in FGM compared to those from the western region. In terms of ethnicity, the tribes that predicted FGM when compared with the Akan tribe were Guan, Gruma, Mole-Dagbani, Grus, Mand, and other tribes. This confirms similar study results in which FGM practice is highly predominant among women in the Upper West Region and the Mole/Dagbani [14]. Ethnicity brings cultural variation, and it is believed the variable nature of people and their culture contributes to the ethnic variations [5].

Women with at least a primary educational level were less likely to engage in FGM compared to those with no education; meaning, lack of formal education was a predictive factor for FGM. Additionally, women from the richest wealth index quintile were more likely 1.8 times to engage in FGM compared to those from the poorest wealth index quintile. In sub-Saharan African studies, economic status had a significant relation with FGM, and not in line with this current study, women from better economic homes were less likely to involve in FGM. [10, 11]. However, in line with this current study, well-educated women were less likely to practice FGM [10–13].

The history of FGM was less likely 0.8 times among women with a marriage age of 15-24 years compared to those with marriage age below 15 years. The implication of this is that child marriage below 15 years was more likely among those with FGM practice history, and this is in line with other earlier studies or works that FGM had a significant relation with child marriage below 18 years [15, 16].

## 5. Conclusion

The study prevalence of FGM is still high in the northern part of Ghana, and the predicted factors were residence region, ethnicity, educational level, and economic status.

## Figures and Tables

**Table 1:** Socioeconomic characteristics of the respondents.

		Frequency	Percentage
Area	Urban	4703	43.4%
	Rural	6137	56.6%
Region	Western	1036	9.6%
	Central	905	8.3%
	Greater Accra	1117	10.3%
	Volta	659	6.1%
	Eastern	820	7.6%
	Ashanti	1693	15.6%
	Brong-Ahafo	914	8.4%
	Northern	1151	10.6%
	Upper East	1146	10.6%
	Upper West	1399	12.9%
Age at first marriage	Below 15	1237	11.9%
	15-24	7611	73.1%
	25-34	1435	13.8%
	35 years and above	128	1.2%
Ethnicity	Akan	4035	37.2%
	GA/Dangme	666	6.1%
	Ewe	900	8.3%
	Guan	362	3.3%
	Gruma	458	4.2%
	Mole Dagbani	2976	27.5%
	Grusi	460	4.2%
	Mande	67	0.6%
	Other	910	8.4%
Educational level	Preprimary or none	4101	37.8%
	Primary	1994	18.4%
	JSS/JHS/middle	3370	31.1%
	SSS/SHS/secondary	939	8.7%
	Higher	434	4.0%
Have health insurance	Yes	6030	55.6%
	No	4810	44.4%
Functional difficulties (age 18-49 years)	Has functional difficulty	1214	11.2%
	Has no functional difficulty	9607	88.8%
Wealth index quintile	Poorest	3217	29.7%
	Second	1791	16.5%
	Middle	1878	17.3%
	Fourth	1909	17.6%
	Richest	2045	18.9%

**Table 2:** Prevalence of female genital mutilation in Ghana.

		Circumcised			
		No		Yes	
Region	Western	992	95.8%	44	4.2%
	Central	897	99.1%	8	**0.9%**
	Greater Accra	1087	97.5%	28	2.5%
	Volta	654	99.2%	5	0.8%
	Eastern	812	99.1%	7	**0.9%**
	Ashanti	1617	95.6%	75	4.4%
	Brong-Ahafo	876	95.8%	38	4.2%
	Northern	1065	92.5%	86	7.5%
	Upper East	871	76.0%	275	24.0%
	Upper West	693	49.5%	706	**50.5%**
Total		9564	88.3%	1272	**11.7%**

**Table 3:** Association socioeconomic characteristics and female genital mutilation in Ghana.

		Circumcised		*X* ^2^	Df	*P* value
		No	Yes			
Area	Urban	4548	151	582.021	1	≤ .001
	Rural	5016	1121			
Region	Western	992	44	2770.331	9	≤ .001
	Central	897	8			
	Greater Accra	1087	28			
	Volta	654	5			
	Eastern	812	7			
	Ashanti	1617	75			
	Brong-Ahafo	876	38			
	Northern	1065	86			
	Upper East	871	275			
	Upper West	693	706			
Age at first marriage	Below 15	964	273	158.316	3	≤ .001
	15-24	6722	885			
	25-34	1339	96			
	35 years and above	118	10			
Ethnicity	Akan	4021	14	1963.652	3	≤ .001
	GA/Damgme	662	2			
	Ewe	897	3			
	Guan	353	9			
	Gruma	444	14			
	Mole Dagbani	2073	902			
	Grusi	325	135			
	Mande	46	21			
	Other	737	172			
Educational level	Preprimary or none	3060	1041	1217.089	4	≤ .001
	Primary	1859	135			
	JSS/JHS/middle	3299	67			
	SSS/SHS/secondary	913	26			
	Higher	431	3			
Have health insurance	Yes	5420	608	35.802	1	≤ .001
	No	4144	664			
Functional difficulties (age 18-49 years)	Has functional difficulty	1063	151	.608	1	.436
	Has no functional difficulty	8482	1121			
Wealth index quintile	Poorest	2384	833	990.454	1	≤ .001
	Second	1574	217			
	Middle	1761	115			
	Fourth	1857	51			
	Richest	1988	56			

**Table 4:** Multiple regression for predictors of female genital mutilation in Ghana.

	B	S.E.	Wald	Sig.	AOR	95% C.I. for AOR	
						Lower	Upper
Residence (rural/urban)	.831	.138	36.408	≤ .001	2.296	1.753	3.007
Western			403.536	≤ .001			
Central	-.852	.424	4.039	.044	.426	.186	.979
Greater Accra	-.317	.301	1.115	.291	.728	.404	1.312
Volta	-1.762	.544	10.489	.001	.172	.059	.499
Eastern	-1.746	.440	15.784	≤ .001	.174	.074	.413
Ashanti	-.692	.232	8.917	.003	.500	.318	.788
Brong-Ahafo	-1.193	.265	20.245	≤ .001	.303	.180	.510
Northern	-1.569	.229	46.909	≤ .001	.208	.133	.326
Upper East	-.513	.212	5.846	.016	.599	.395	.907
Upper West	.608	.206	8.700	.003	1.836	1.226	2.750
Below 15			9.009	.029			
15-24	-.284	.095	8.845	.003	.753	.624	.908
25-34	-.247	.158	2.429	.119	.782	.573	1.066
35 years and above	-.062	.435	.020	.887	.940	.401	2.206
Akan			246.865	≤ .001			
GA/Dangme	-.038	.772	.002	.961	.963	.212	4.373
Ewe	.364	.660	.304	.581	1.439	.395	5.240
Guan	2.188	.472	21.465	≤ .001	8.919	3.534	22.509
Gruma	1.864	.407	20.988	≤ .001	6.447	2.905	14.310
Mole Dagbani	3.640	.299	148.010	≤ .001	38.100	21.195	68.489
Grusi	3.813	.314	147.261	≤ .001	45.302	24.470	83.869
Mande	4.228	.408	107.642	≤ .001	68.576	30.853	152.421
Other	3.379	.306	122.185	≤ .001	29.328	16.111	53.388
Preprimary or none			136.214	≤ .001			
Primary	-.712	.111	40.934	≤ .001	.491	.395	.610
JSS/JHS/middle	-1.363	.148	84.693	≤ .001	.256	.191	.342
SSS/SHS/secondary	-1.211	.232	27.142	≤ .001	.298	.189	.470
Higher	-2.625	.606	18.773	≤ .001	.072	.022	.238
Health insurance	.109	.075	2.077	.150	1.115	.962	1.293
Poorest			14.105	.007			
Second	.079	.104	.568	.451	1.082	.882	1.327
Middle	-.019	.143	.018	.892	.981	.741	1.298
Fourth	-.223	.194	1.329	.249	.800	.547	1.169
Richest	.587	.211	7.718	.005	1.799	1.189	2.722

## Data Availability

All data related to the findings of this study are available from the Multiple Indicator Cluster Survey (MICS) website upon request.
